# A network biology approach to unraveling inherited axonopathies

**DOI:** 10.1038/s41598-018-37119-z

**Published:** 2019-02-08

**Authors:** Dana M. Bis-Brewer, Matt C. Danzi, Stefan Wuchty, Stephan Züchner

**Affiliations:** 1 Dr. John T. Macdonald Foundation Department of Human Genetics, John P. Hussman Institute for Human Genomics, University of Miami Miller School of Medicine, Miami, Florida USA; 2 Department of Computer Science and Department of Biology, University of Miami, Coral Gables, Florida USA

## Abstract

Inherited axonopathies represent a spectrum of disorders unified by the common pathological mechanism of length-dependent axonal degeneration. Progressive axonal degeneration can lead to both Charcot-Marie-Tooth type 2 (CMT2) and Hereditary Spastic Paraplegia (HSP) depending on the affected neurons: peripheral motor and sensory nerves or central nervous system axons of the corticospinal tract and dorsal columns, respectively. Inherited axonopathies display an extreme degree of genetic heterogeneity of Mendelian high-penetrance genes. High locus heterogeneity is potentially advantageous to deciphering disease etiology by providing avenues to explore biological pathways in an unbiased fashion. Here, we investigate ‘gene modules’ in inherited axonopathies through a network-based analysis of the Human Integrated Protein-Protein Interaction rEference (HIPPIE) database. We demonstrate that CMT2 and HSP disease proteins are significantly more connected than randomly expected. We define these connected disease proteins as ‘proto-modules’ and show the topological relationship of these proto-modules by evaluating their overlap through a shortest-path based measurement. In particular, we observe that the CMT2 and HSP proto-modules significantly overlapped, demonstrating a shared genetic etiology. Comparison of both modules with other diseases revealed an overlapping relationship between HSP and hereditary ataxia and between CMT2 + HSP and hereditary ataxia. We then use the DIseAse Module Detection (DIAMOnD) algorithm to expand the proto-modules into comprehensive disease modules. Analysis of disease modules thus obtained reveals an enrichment of ribosomal proteins and pathways likely central to inherited axonopathy pathogenesis, including protein processing in the endoplasmic reticulum, spliceosome, and mRNA processing. Furthermore, we determine pathways specific to each axonopathy by analyzing the difference of the axonopathy modules. CMT2-specific pathways include glycolysis and gluconeogenesis-related processes, while HSP-specific pathways include processes involved in viral infection response. Unbiased characterization of inherited axonopathy disease modules will provide novel candidate disease genes, improve interpretation of candidate genes identified through patient data, and guide therapy development.

## Introduction

The inherited axonopathies spectrum represents a group of disorders unified by length-dependent axonal degeneration. Progressive axonal degeneration can lead to both Charcot-Marie-Tooth type 2 (CMT2) and hereditary spastic paraplegia (HSP) depending on the affected neurons: peripheral motor and sensory nerves or central nervous system axons of the corticospinal tract and dorsal columns, respectively. Though CMT2 and HSP have been historically classified as two separate disorders, divided by peripheral vs central nerve axons, they are now studied together as a spectrum of inherited axonopathies based on their increasingly apparent clinical and genetic overlap^1–3^. Charcot-Marie-Tooth (CMT) disease is the most common inherited neurological disorder with an estimated prevalence of 4 to 8 per 10,000^4–7^. CMT typically causes distal muscle weakness and atrophy, high arched feet, decreased tendon reflexes, and sensory loss resulting from progressive polyneuropathy of the motor and/or sensory nerves^4,8^. Hereditary spastic paraplegias (HSPs) cause bilateral lower limb spasticity and weakness^9^. HSPs have a prevalence of 1.2–9.6 per 100,000^9^.

Since the advent of next generation sequencing, Mendelian disease gene discovery has made notable progress with over 80 identified CMT2 and over 90 HSP genes^10–12^. At least five of these genes have been shown to cause either CMT2, HSP, or a mixed phenotype, depending on the underlying mutation^1^. The functional characterization of inherited axonopathy disease genes has improved our understanding of the affected biological mechanisms^2,3,13^. Evidently, the pathogenic molecular mechanisms of CMT2 and HSP overlap, including disruption of axonal transport, mitochondrial dynamics, mitochondrial regulation, membrane trafficking, and organelle shaping^14^. These functional similarities cohere as the long axons of the upper and lower motor neurons require adaptable cellular machinery to distribute molecules, maintain neuronal homeostasis, and create a specialized neuronal cytoskeleton^3^.

Inherited axonopathies display an extreme degree of genetic heterogeneity^3^. High locus heterogeneity is advantageous to studies of disease etiology by providing avenues to explore biological pathways through a network biology approach. These approaches focus on the complex network of functional interdependencies between cellular components in human disease^15^. In extension, network medicine is based on the hypothesis that the impact of a single mutated gene is propagated along the gene’s network links^15^. The links between a set of genes can be summarized into a disease module. Identification and characterization of such disease modules will help resolve differences between normal and disease pathways, reveal common biological functions in related diseases, and identify novel disease gene candidates and pathways related to disease^15–18^. As an example, a recent protein interaction network analysis of HSP genes identified three candidate genes, which were subsequently found to be mutated in HSP families^19^. Finally, it is unlikely that traditional pharmacological approaches will develop a drug for every single CMT and HSP gene, but rather aim at influencing key gene networks.

Here, we investigate the inherited axonopathy disease modules through an unbiased analysis of protein-protein interactions, provided by the Human Integrated Protein-Protein Interaction rEference (HIPPIE), with the objectives of determining the topological relationship between inherited axonopathies and defining and characterizing their putative disease modules^20^. We evaluate the overlap between disease modules with a shortest path-based measurement^21^ and observe that the CMT2 and HSP proto-modules significantly overlapped, demonstrating a shared genetic etiology. We expand each disease module by incorporating candidate genes with significant connections to disease-related seed genes^17^. Such disease modules indicate an enrichment of ribosomal proteins and pathways, likely involved in inherited axonopathy pathogenesis. Our results suggest that unbiased characterization of inherited axonopathy disease modules provide novel candidate disease genes, improve interpretation of candidate genes identified through patient data, and guide therapy development.

## Materials and Methods

### Disease Genes

Charcot-Marie-Tooth type 2 (CMT2) and Hereditary Spastic Paraplegia (HSP) input-proteins, also known as “seed protein”, were selected through an extensive literature review by domain experts from the Rare Diseases Clinical Research Network (RDCRN). Specifically, the CMT2 gene list was obtained from the Inherited Neuropathy Consortium and the HSP gene list was obtained from the Alliance for Treatment in HSP and PLS, and the Clinical Research in ALS and Related Disorders for Therapeutic Development consortium. Non-syndromic CMT2 genes (n = 65) and both complex and pure HSP genes (n = 96) were included as seed proteins for disease module construction. To evaluate the topological distance between CMT2 and HSP in a broader context, we selected seven additional diseases for topological separation analysis. We chose diseases that vary in degree of expected shared etiology with inherited axonopathies. Such considerations are based on clinical symptoms and shared disease genes, with hereditary ataxia expected to be the closest and cancer expected to be the most distance diseases. Seed proteins for comparison of diseases (cancer (CA), deafness (DFN), cardiomyopathy (CM), muscular dystrophy (MD), Parkinson’s disease (PD), amyotrophic lateral sclerosis (ALS), and hereditary ataxia (ATX)) were collected from the Online Mendelian Inheritance in Man (OMIM, updated 10-25-2017)^22^. Supplementary Table 1 contains all seed proteins used in this study, and Supplementary Fig. 1A displays the degree distribution for seed proteins.

### Molecular Interactions

The Human Integrated Protein-Protein Interaction rEference (HIPPIE) is a publically available, scored protein-protein interactions (PPI) dataset^23^. HIPPIE integrates data from BioGRID (genetic interactions removed), DIP, HPRD, IntAct, MINT, BIND, and MIPS databases. HIPPIE provides a curated scoring scheme that is based on the experimental technique to identify the PPI, the number of studies that have reported the PPI, and the reproducibility of the PPI across model organisms. The HIPPIE confidence score ranges from 0 to 1 and reflects the quality of experimental evidence supporting each PPI. The HIPPIE authors have predefined confidence levels based on the quartiles of all confidence scores: medium confidence (0.63 – second quartile of the HIPPIE score distribution) or high confidence (0.73 – third quartile) (http://cbdm-01.zdv.uni-mainz.de/mschaefer/hippie/information.php#sources). To balance network coverage and reasonable interaction confidence, we accounted for the interactions with at least a medium confidence score of 0.63. The most recent update includes approximately 273,900 experimentally determined PPIs between 17,000 human proteins^20^. The HIPPIE (v2.0) dataset was pre-filtered to remove interactions from non-human sources and below the predefined medium-confidence score threshold in order to create a more stringent global interactome of 204,215 interactions between 15,690 proteins. The filtered HIPPIE protein-protein interaction network thus obtained is scale-free, as demonstrated by a power-law decay in the corresponding degree distribution (Supplementary Fig. 1B).

### Disease Proto-Module Construction

A goal of network analysis is to define and study a disease module. We first define a disease proto-module since our knowledge of both the disease-causing proteins and all human protein interactions is incomplete^21^. Disease proto-modules are considered clusters of highly connected seed proteins that were constructed by retrieving the subgraph of directly connected seed proteins from the filtered HIPPIE network. The connectivity of a proto-module is quantified by the size of the largest connected component (LCC) and shortest distance (SD) between proteins^21^. While LCC indicates the number of directly connected disease proteins, SD measures the localization of disease proteins that do not directly interact. Utilizing publically available Python scripts, we calculate the LCC, SD, and significance of each disease proto-module^21^. To assess the significance of LCCs, expected null distributions were calculated by randomizing sets of proteins of equal seed list size. We determined the expected distribution of LCCs after 10,000 randomizations and calculated a proto-module specific *z*-score^21^. As previously described, a proto-module is obtained if the corresponding *z*-score was ≥1.6 with a significance *p*-value ≤ 0.05^21^. To assess the SDs, the distribution of SDs after 10,000 randomizations was tested using a Mann-Whitney-U test^21^.

### Disease Proto-Module Expansion

The DIseAse MOdule Detection (DIAMOnD) algorithm is one of the latest and most utilized disease-gene prediction methods^17,24^. We selected the highly cited DIAMOnD algorithm for this study based on the previous successful application to a diverse range of diseases^18,25–28^. Specifically, DIAMOnD expands each proto-module to identify the putatively complete disease module^17^. Based on the assumption that disease proteins interact more significantly with each other, DIAMOnD explores the topological neighborhood of the seed proteins based on the significance of connections to disease proteins^17^. Briefly, DIAMOnD calculates the connectivity significance for all proteins that interact with the seed proteins; ranks the proteins according to *p*-values; incorporates the highest ranked protein into the set of seed proteins; and repeats the process to expand the disease seed set^17^. Each disease proto-module was expanded using DIAMOnD recommended settings, including weighting the seed proteins to 10 and running 200 iterations. The core inherited axonopathy module was constructed from the intersection of the CMT2 and HSP expanded modules.

### Disease Proto-Module Separation

The proximity of diseases within a network is useful for understanding their biological and clinical overlap. The network-based separation between two disease proto-modules *A* and *B* was assessed by comparing the mean shortest distances of seed proteins within each proto-module (*d*_AA_ and *d*_BB_) to the mean shortest distance between the proteins of each proto-module (*d*_AB_)^21^. In particular, Menche *et al*. defined the network-based separation as $d{\rm{AB}}-\frac{d{\rm{AA}}+d{\rm{BB}}}{2}$ and calculated a z-score to quantify the difference between the observed separation and random expectation and analytically calculate a corresponding *p*-value for each *z*-score^21^. A positive *z*-score indicates separation of disease pairs while a negative *z*-score indicates overlap. The |*z*-score| must be ≥1.6 for a disease pair to be more/less overlapping than randomly expected with a significance *p*-value ≤ 0.05^21^.

### Semantic Similarity Within Expanded Module

The biological functions of the expanded modules were assessed with Gene Ontology (GO) semantic similarity measurements using the GOSemSim R package^29–31^. The semantic similarity was computed using the graph-based Wang method, which computes the semantic similarities between terms based on both their topology within the GO graph structure and their relations with ancestor terms^31^. Semantic similarities of multiple GO terms were combined using the Best-Match Average (BMA) strategy which calculates the average of all maximum similarities on each pair of GO terms^31^. A systematic evaluation of the metrics for GO based semantic similarity found that the BMA is the best combination approach since it yields the highest resolutions and does not show the undesired behaviors of the maximum approach or the average approach^32^. Before computing the semantic similarities of GO terms, we filtered the GO terms based on annotation evidence codes to produce a high quality dataset. Specifically, we removed Inferred from Electronic Annotation (IEA) evidence codes. Although IEA are the most common evidence codes, they also represent weak associations as they are based on electronic curation without human curation. Since the input for this analysis (DIAMOnD proteins) is based on a dataset of physical interactions, we also removed the Inferred from Physical Interaction (IPI) evidence codes to remove bias and circular reasoning from the analysis.

### Functional Enrichment Analysis of Disease Module

Functional enrichment analysis was performed for GO Biological Process (BP) terms, Kyoto Encyclopedia of Genes and Genomes (KEGG) pathways, and Wikipathways^29,33,34^. Over-Representation Analysis (ORA) of protein clusters, based on GO BP semantic similarity were analyzed using the DAVID Functional Annotation Tool^35^. ORA of disease modules for KEGG pathways and Wikipathways was performed using WebGestalt and were filtered for FDR ≤ 0.2^36^. Pathway ORA was performed on 6 sets: the expanded CMT2 module, the expanded HSP module, the intersection of the expanded CMT2 and HSP modules (AXONO core module), the union of the expanded CMT2 and HSP modules (AXONO spectrum module), the difference of the expanded CMT2 and HSP modules (CMT2-only module), and the difference of the HSP and CMT2 modules (HSP-only module). Because of gene redundancy in pathway sets, the Cytoscape plugin Enrichment Map was used to visualize the similarity between enriched pathway sets with a Jaccard coefficient greater than 0.1^37^.

## Results

### Constructing the inherited axonopathy modules

Disease module construction requires an input seed list of disease proteins and a molecular interaction network. We curated 65 non-syndromic axonal Charcot-Marie-Tooth 2 (CMT2) and 96 Hereditary Spastic Paraplegia (HSP) input seed genes through an extensive literature review (Supplementary Table 1). As a protein-protein interaction data source, we used the Human Integrated Protein-Protein Interaction rEference (HIPPIE, v2.0) that integrates interaction data from high quality databases and provides a high-performing scoring system^20^. The HIPPIE confidence score is based on the amount and the reliability of supporting experimental evidence for each interaction^23^. Applying the predefined medium confidence score (0.63 – second quartile of the HIPPIE score distribution), we created a medium-confidence global interactome consisting of 204,215 interactions between 15,690 human proteins.

**Figure 1:**
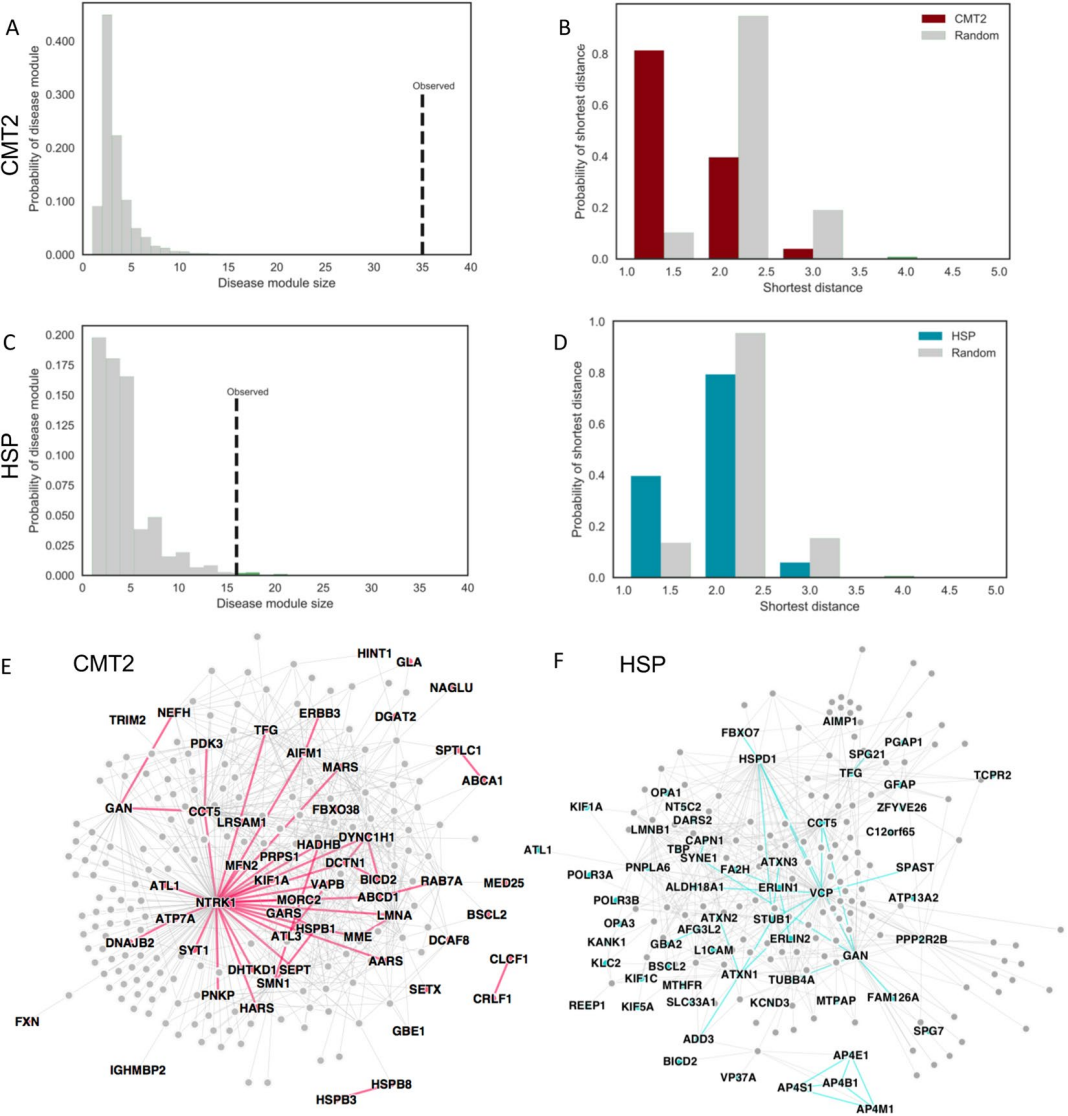
Inherited axonopathy proto-module connectivity (**A**–**D**) and expanded module networks (**E**,**F**). (**A**,**B**) Increased observed largest connected component (LCC) size of CMT2 (**A**) and HSP (**B**) disease seed genes in comparison to the expected, random distribution. (**C**,**D**) Increased shortest distance between inherited axonopathy disease genes in comparison to the expected, random expectation. (**E**,**F**) First degree interactions between known Mendelian disease genes (colored) and DIAMOnD genes (gray) for CMT2 (**E**) and HSP (**F**). Disease specific genes are labeled, and direct edges between disease genes are more heavily weighted.

In an interactome, disease associated proteins are expected to cluster into specific network neighborhoods through physical interactions^15,38^. The inherited axonopathy proteins follow this expectation by forming significantly connected disease modules (Fig. 1). The largest connected component (LCC) of a subgraph measures the number of seed proteins in the largest subgraph composed of direct interactions between seed proteins. In our analysis, the CMT2 and HSP LCCs are significantly larger than expected by random chance (CMT2: *n* = 35, *z-*score = 17.1; HSP: *n* = 16, *z-*score = 4.0) (Fig. 1A,C), indicating that the CMT2 and HSP disease proteins form disease proto-modules composed of proteins with high biological and functional similarity. Another measure of connectivity is the shortest distance (SD) between seed proteins. SD also includes fragmented seed proteins that do not connect to proteins in the LCC, providing a more global measure of connectivity than LCC alone. As the knowledge of human protein interactome and disease etiology is incomplete, seed proteins are expected to fragment within the interactome, pointing to larger distances. However, we observed that SD distributions of CMT2 and HSP seed genes are shifted towards 1.0, indicating direct connections between seed proteins. In comparison to the expected distribution based on randomized protein sets (Fig. 1B,D) the observed means are significantly smaller (Mann Whitney U Test; CMT2: *p*-value = 2.7E-16; HSP: *p*-value = 5.0E-05). Taken together, the significant connectivity measurements demonstrate that the CMT2 and HSP disease proteins form agglomerations that can be expanded and further studied (Fig. 1).

The significantly connected CMT2 and HSP proto-modules were next used as seed modules to expand into putatively “complete” disease modules using the DIseAse Module Detection (DIAMOnD) method (Supplementary Table 2)^17^. The DIAMOnD algorithm identifies the topological neighborhood of the seed proteins based on the significance of connections to seed proteins and has been successfully used to study asthma and cardiovascular endophenotypes^18,25^. After performing 200 iterations of the DIAMOnD method, an additional 53 CMT2 proteins and 61 HSP proteins were incorporated into each LCC, which were originally fragmented and not directly connected (Fig. 1E,F). To limit the incorporation of false positives into each inherited axonopathy module, the DIAMOnD proteins were evaluated for biological evidence by comparing the Gene Ontology Biological Process (GO BP) terms of the DIAMOnD proteins to the GO BP terms of the known disease proteins. The GO BP terms were compared by hierarchical clustering based on semantic similarity (Supplementary Fig. 2)^29^, allowing us to infer the biological relationship between DIAMOnD proteins and disease proteins. As indicated by the semantic similarity clustering, DIAMOnD proteins are functionally related to the disease seed proteins, suggesting that we limited the incorporation of false positives beyond the true biological limit. To understand the underlying biological function, we performed GO BP Over Representation Analysis (ORA) of the proteins within each cluster. In particular, we confirmed known cellular processes including axonal transport, microtubule dysregulation, and mitochondrial involvement (Supplementary Fig. 2). Taken together, these results indicate that the expansion of proto-modules leads to the identification of proteins that are functionally similar to disease proteins and are involved in biological processes that are relevant to the disease. After topologically validating the disease modules with connectivity measures and biologically validating the incorporated proteins through GO BP analysis, we present the final CMT2 and HSP disease modules in Fig. 1E,F. The networks display the connections between disease proteins (in color) and DIAMOnD proteins (in gray). The DIAMOnD proteins (Supplementary Table 2) will improve our understanding of the disease by adding power to pathway analysis and containing new candidate disease genes and targets.

### Topological relationship of disease modules

**Figure 2:**
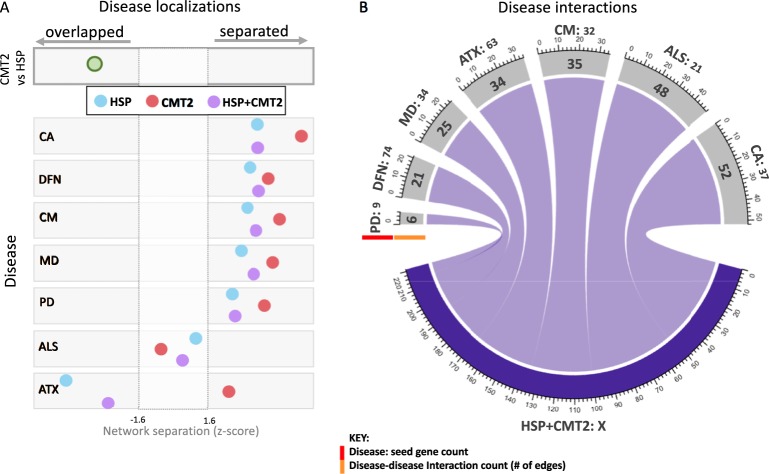
Disease proto-modules separation and connections. (**A**) Significance of topological relationships between axonopathy proto-modules (Charcot-Marie-Tooth type 2 only: CMT2; Hereditary Spastic Paraplegia only: HSP; combined HSP + CMT2) and control diseases (Cancer: CA, Deafness: DFN, Cardiomyopathy: CM, Muscular Dystrophy: MD, Parkinson’s Disease: PD, Amyotrophic Lateral Sclerosis: ALS, and Hereditary Ataxia: ATX). Gray dotted lines indicate the threshold for significance (|Z-scores| >=1.6). Negative (positive) Z-scores indicate overlapped (separated) modules. (**B**) Direct connections between HSP + CMT2 disease proteins and comparison disease proteins. Disease names and input seed protein count (highlighted by red bar) for each disease compared are indicated outside the circle. Direct interactions between disease proteins of HSP + CMT2 are displayed on the outer gray bar (highlight by orange bar).

Disease modules that are located in adjacent network areas will likely share proteins, interactions, and pathways involved in disease pathogenesis^21^. Disruption of overlapping functional modules will likely result in shared clinical characteristics in each disease^21^. The topological relationship between inherited axonopathy proto-modules (i.e. CMT2, HSP) was quantified through the shortest distances between modules (Fig. 2). The close relationship within the spectrum of inherited axonopathies (between CMT2 and HSP) is confirmed in our analysis by significantly overlapping disease proto-modules (network distance = −0.15; *z*-score = −2.6) (Fig. 2A). We next explored the relationships of the inherited axonopathies with cancer (CA), deafness (DFN), cardiomyopathy (CM), muscular dystrophy (MD), Parkinson’s disease (PD), amyotrophic lateral sclerosis (ALS), and hereditary ataxia (ATX) (Fig. 2A and Supplementary Fig. 3). Utilizing disease genes curated from the OMIM database (updated 10-25-2017), we compared the corresponding disease-specific proto-modules with CMT2, HSP, and their union (HSP&CMT2). All compared diseases were significantly separated from each axonopathy module except for ALS and ATX. Notably, the ALS proto-module was not significantly separated nor overlapped with the CMT2, HSP, and HSP&CMT2 proto-modules. The ATX proto-module overlapped significantly with both the HSP and HSP&CMT2 proto-modules, yet significantly separated from the CMT2 proto-module. Since the network separation parameter depends on the number of proteins in each disease and the number of direct interactions between disease proteins, we provided the count of seed proteins (ranging from 9 to 72) and the amount of direct connections between disease proteins (ranging from 6 to 52 connections) (Fig. 2B).

### Ribosomal proteins are enriched within the inherited axonopathy modules

**Figure 3:**
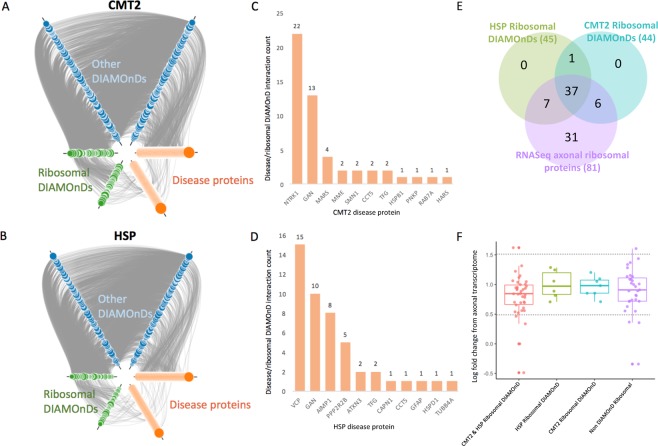
Interactions within the expanded inherited axonopathy modules. (**A**,**B**) First degree interactions within CMT2 (**A**) and HSP (**B**) expanded modules. DIAMOnD proteins are separated into ribosomal proteins (green) and other (blue). Size of DIAMOnD nodes are proportional to DIAMOnD order of incorporation – smaller nodes were incorporated earlier. (**C**,**D**) First degree interaction counts between disease protein and ribosomal DIAMOnD proteins. (**E**) Relationships between HSP ribosomal DIAMOnD proteins, CMT ribosomal DIAMOnD proteins, and ribosomal proteins significantly localized to the axons of human motor neurons (*q*-value ≤ 0.1) (**F**). Distribution of log fold changes between axonal and soma differential localization.

The CMT2 and HSP expanded modules both contained an unexpected number of ribosomal proteins incorporated as DIAMOnD proteins (CMT2 *n* = 47; HSP *n* = 48; overlap *n* = 40) (Fig. 3). To qualitatively determine if the enriched ribosomal proteins are an artifact of the global network structure, we examined the underlying protein interactions (Fig. 3A,B). The interactions between ribosomal DIAMOnD proteins and disease seed proteins (green nodes connecting to orange nodes) are similar to the interactions between disease seed proteins (orange nodes connecting to orange nodes), suggesting true biological interactions instead of a topology-biased artifact. We then quantified the direct interactions between disease seed proteins and ribosomal DIAMOnD proteins - CMT2: minimum: 1, maximum: 22, mean: 4.6; HSP: minimum: 1, maximum: 15, mean: 4.2 (Fig. 3C,D). To further verify that ribosomal protein interactions are not an artifact of the HIPPIE network, we expanded each comparison disease proto-module and quantified the first-degree interactions between disease seed proteins and ribosomal DIAMOnD proteins (Supplementary Figure 4). We found that neurologically related diseases contained more ribosomal DIAMOnD proteins than the non-neurological control disease, demonstrating disease-specific enrichment. Furthermore, in a recent study of the human motor neuron transcriptome, 81 ribosomal proteins showed significant localization (*q*-value ≤ 0.1) to the axon with modest expression levels (median = 0.89) (Fig. 3E,F)^39^. In the Venn diagram in Fig. 3E, we observed that 50 of the axonal ribosomal DIAMOnDs were significantly localized to the axon, with 37 of these present in both the CMT2 and HSP sets of DIAMOnDs. In Fig. 3F, we show that the distributions of expression level log fold changes (LFCs) of the ribosomal DIAMOnDs (shared CMT2-HSP: n = 37, HSP only: n = 7, CMT2 only: n = 6) and the ribosomal non-DIAMOnDs (n = 31) were highly similar, corroborating the biological relevance of the network analysis results. Eight ribosomal proteins were reported as differentially localized to the axons in comparison to the soma (*q*-value ≤ 0.1; LFC ≤ 0.05 or ≥1.5)^39^. RPL26, RPL28, RPL7A, and RPS6KA1 are DIAMOnD proteins while RPL31, RPL7L1, RPS6KC1, and RSL24D1 are not (Fig. 3F). Significant axonal localization of 50 ribosomal DIAMOnD proteins demonstrates a biological significance, with potential to explain the enrichment of ribosomal proteins within neurologically-related disease network.

### Pathway analysis of the inherited axonopathy spectrum modules

**Figure 4:**
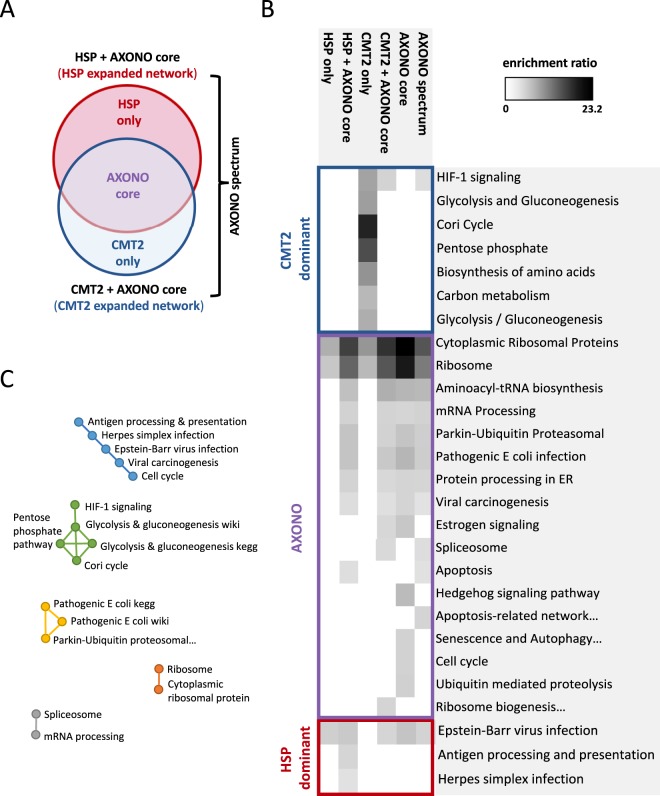
Pathway analysis of inherited axonopathy modules. (**A**) Venn diagram of disease gene sets used for pathway enrichment including the expanded CMT2 module, the expanded HSP module, the intersection of the expanded CMT2 and HSP modules (AXONO core), the union of the expanded CMT2 and HSP modules (AXONO spectrum), the difference of the expanded CMT2 and HSP modules (CMT2-only), and the difference of the HSP and CMT2 modules (HSP-only). (**B**) KEGG and WikiPathways significantly enriched within each disease module set. (**C**) Closely related pathways based on redundancy in proteins involved in each pathway.

Functional analysis of the inherited axonopathy spectrum modules was performed by an Over-Representation Analysis (ORA) of WikiPathways and Kyoto Encyclopedia of Genes and Genomes (KEGG) pathways (Supplementary Table 3)^33,34^. Our results show both unique and shared pathways across inherited axonopathies (Fig. 4). Pathway enrichment analysis was performed on 6 sets of proteins: the expanded CMT2 module (*n* = 254 proteins), the expanded HSP module (*n* = 295 proteins), the intersection of the expanded CMT2 and HSP modules (AXONO core module, *n* = 152 proteins), the union of the expanded CMT2 and HSP modules (AXONO spectrum module, *n* = 397 proteins), the difference of the expanded CMT2 and HSP modules (CMT2-only module, *n* = 102 proteins), and the difference of the HSP and CMT2 modules (HSP-only module, *n* = 143 proteins) (Fig. 4A). We simplified the 6 protein sets to 3 protein sets for pathway classification and visualization purposes and accounted for the CMT2-dominant, HSP-dominant sets and the intersection of CMT2-expanded and HSP-expanded networks (AXONO). We assigned each enriched pathway to the most influenced disease module (CMT2-dominant, HSP-dominant, or AXONO) based on the combination of module sets that was enriched for a pathway (Fig. 4B). For example, pathways that were enriched in the CMT2 only set, but not in the HSP only set, were categorized as a CMT2-dominant pathway. The pathway results can be ranked by the highest count of observed input proteins within the pathway set or the highest enrichment score within the pathway set, each averaged across the 6 disease module sets. The Cytoplasmic Ribosomal Proteins (wiki) and Ribosome (KEGG) pathways were the most highly enriched with the highest count of input proteins within each module set, likely due to the high amount of ribosomal DIAMOnD proteins. Excluding the ribosomal protein pathways, the top 5 pathways (based on the observed count of input proteins) are: broad axonopathy spectrum: mRNA Processing, Protein processing in the endoplasmic reticulum, Viral carcinogenesis, Spliceosome, and Apoptosis; CMT2-dominant: HIF-1 signaling pathway, Aminoacyl-tRNA biosynthesis, Biosynthesis of amino acids, Carbon metabolism, and Glycolysis and Gluconeogenesis; and HSP-dominant: Epstein-Barr virus infection, Herpes simplex infection, and Antigen processing and presentation. The top 5 most enriched pathways, excluding the ribosomal protein pathways, are: broad axonopathy spectrum: Aminoacyl-tRNA biosynthesis, Hedgehog signaling pathway, Pathogenic Escherichia coli infection, Parkin-Ubiquitin Proteasomal System pathway, and Estrogen signaling pathway; CMT2-dominant: Cori Cycle, Pentose phosphate pathway, Biosynthesis of amino acids, Glycolysis and Gluconeogenesis, and Carbon metabolism; and HSP-dominant: Epstein-Barr virus infection, Antigen processing and presentation, and Herpes simplex infection. The enriched pathways were simplified for easier understanding by grouping pathways together based on overlapping proteins (Fig. 4C).

## Discussion

The goal of this study was to compare the spectrum of inherited axonopathies by identifying and characterizing the disease modules of axonal Charcot-Marie-Tooth disease (CMT2) and Hereditary Spastic Paraplegia (HSP). To our knowledge, this is the first study to apply an unbiased network analysis approach to CMT2 or inherited axonopathies combined.

### Inherited axonopathies form significantly connected disease modules

Disease genes are not distributed randomly within biological interaction networks but form local connections with each other as they interact to perform related biological functions^18,38,40^. We began our analysis by identifying these localized gene neighborhoods of inherited axonopathy proteins. Since the genetic etiology of inherited axonopathies is not completely known, our analysis is limited by incomplete input seed lists. However, we found that CMT2 and HSP genes were significantly more connected to each other than expected by chance (Fig. 1A–D). The identification of these significant disease-specific proto-modules highlights the robustness of network analysis (Fig. 1E,F).

Overlapping disease modules are expected to share clinical characteristics, pointing to physical interaction of proteins, and by extension, common biological pathways^21^. Since CMT2 and HSP have been categorized as inherited axonopathies based on their clinical and genetic overlap, we anticipated and observed a significant overlap between CMT2 and HSP disease proto-modules within the network (Fig. 2A). We found a significant separation of CMT2, HSP, and BOTH proto-modules from cancer, deafness, cardiomyopathy, muscular dystrophy, and Parkinson’s disease; an unclear relationship with amyotrophic lateral sclerosis; and discordant distances to ataxia (Fig. 2A). A previous study constructed an “HSPome” from published and candidate HSP proteins, and observed a significant overlap between HSP with Alzheimer’s disease, Parkinson’s disease (PD), and Amyotrophic Lateral Sclerosis (ALS)^19^. However, in our study, HSP was significantly separated from PD and had an unclear relationship with ALS (Fig. 2A). The difference in topological relationships between studies is likely caused by differences in the underlying data sources and input seed lists. The “HSPome” was created from iRefIndex, HumanNet, and STRING with 43 known HSP proteins as input seed list, whereas we used the HIPPIE database and increased our input seed list to 95 proteins. In particular, 40 seeds overlapped between the two inputs^41–43^. Interestingly, the inherited axonopathy proto-modules appear slightly “less” separated, based on *z*-score, from PD and ALS than the other non-neurological controls (Cancer, Deafness, Cardiomyopathy, and Muscular Dystrophy). Each axonopathy module showed an unclear relationship with ALS (neither significantly overlapped nor separated), which should be clarified in the future with additional seed proteins. Notably, the HSP proto-module is highly overlapping with the ATX proto-module while the CMT2 proto-module is significantly separated. This overlap supports the ataxia-spasticity disease spectrum and warrants further investigation^44^.

### Ribosomal proteins in neurological pathogenesis

The CMT2 and HSP disease modules were identified by expanding the seed protein sets with the recommended 200 iterations of the DIseAse Module Detection (DIAMOnD) method^17^. Exploration of the DIAMOnD proteins revealed that close to 25% are ribosomal proteins (Fig. 3). Ribosomal proteins (RPs) surround a catalytic core of ribosomal RNA (rRNA) to form ribosomes^45,46^. RPs have universal roles in ribosome assembly and function, such as assisting in the proper folding of rRNA^46,47^. Beyond these core roles, RPs have demonstrated extra-ribosomal functions both within the ribosome system, such as regulating and balancing individual RP synthesis with rRNA, and outside of the ribosome system, such as triggering apoptosis in response to disruption in ribosome synthesis^47^. RPs are responsible for various phenotypes across organisms: the *Minute* phenotype in *Drosophila* (delayed development; short, thin bristles; and impaired fertility), gene-specific abnormalities caused by morpholino-induced knock-downs in zebrafish, developmental defects in mice, and 25% of Diamond-Blackfan anemia cases in humans^46,48,49^. Additionally, RPs were shown to be major interactors with kinase substrates of LRRK2 - a familial and sporadic PD protein^50^. Specifically, phosphodeficient ribosomal small protein 15 (RPS15) rescued LRRK2 neurotoxicity by affecting translation^50^. Impaired protein translation has been repeatedly implicated in CMT with mutations within five aminoacyl-tRNA synthetases identified to date^51^. Furthermore, RPs have tissue-specific expression and have been experimentally shown to localize to nerves *in vivo*, including mature peripheral nerve axons and cortical tract axons^52,53^. Locally synthesized RPs can dynamically change specific ribosomal compositions which may allow axons to fine-tune mRNA selectivity^53^. In this study, we show that RPs are appreciable interactors of inherited axonopathy disease proteins. Interestingly, an enrichment of ribosomal proteins was recently observed within the axonal transcriptome from fibroblast-derived human motor neurons (Fig. 3E,F)^39^. Forty-six of the DIAMOnD RPs show modest localization between axon and cell body in a human motor neuron model and an additional four were significantly differential localized to the axon^39^. Furthermore, we observe increased RP involvement in neurological disease controls compared to non-neurological disease (Supplementary Fig. 4). Taken together, our results support the emerging role of translational dysregulation in diverse neurologic diseases. We speculate that RPs may play an important function in neurologic diseases via their extra-ribosomal roles, including alerting the cell to stress^47^.

### Pathway analysis provide insights into inherited axonopathy disease mechanisms

Lastly, we compared the 6 inherited axonopathy spectrum module sets through pathway overrepresentation analysis, and assigned each enriched pathway to the most influenced disease module (CMT2-dominant, HSP-dominant, or AXONO) (Fig. 4; Supplementary Table 3). Since each disease module set contained many ribosomal proteins, the most enriched pathways were the Cytoplasmic Ribosomal Proteins (Wikipathway) and Ribosome (KEGG) pathways. Aside from the ribosomal pathways, we identified pathways known to be involved in inherited axonopathies, such as aminoacyl-tRNA biosynthesis and protein processing in the endoplasmic reticulum (ER). Protein processing in the ER was significantly associated with each module involving the “axonopathy core” set, and is important in both CMT2 and HSP pathophysiology. The ER is the primary site for protein/lipid biosynthesis and intracellular calcium storage, and is tightly coordinated with the mitochondria to maintain the energetically demanding mechanisms of long axons^54^. Many affected axonopathy genes localize to the ER or mitochondria and disrupt the organelles’ coordination^55^. For example, *atlastin-1* can cause both CMT2 and HSP through abnormal ER shaping and mitochondrial transport^3,55^.

Pathways that are not directly implicated in the axonopathy pathogenesis were also identified. The AXONO module was enriched for the Hedgehog signaling pathway and the Estrogen signaling pathway. Sonic hedgehog (Shh) is essential for central nervous system development and is expressed within injured sciatic nerve^56^. Recently, reduced Shh signaling was shown to disturb axon regeneration via misregulated myelin degradation^56^. Estrogen signaling has also been implicated in axon regeneration by accelerating peripheral nerve regeneration and increasing motoneuron participation^57,58^. Another unexpected pathway enriched within the AXONO module was Pathogenic *Escherichia Coli* infection. The *E. Coli* pathways were significantly overlapping with Parkin-Ubiquitin Proteasomal System pathway through the tubulin proteins in each pathway (Fig. 4C). Alteration of the gut microbiome has been suggested to contribute to neurodegenerative diseases, such as Parkinson’s and Alzheimer’s disease^59^. Furthermore, gut inflammation and microbiome alterations may play a role in the development or progression of motor neuron disease and ALS^59–61^.

Distinct pathways were revealed between the CMT2-dominant and HSP-dominant modules. The CMT2-dominant module predominantly involved overlapping glucose metabolism pathways: HIF-1 signaling, glycolysis and gluconeogenesis, Cori cycle, and pentose phosphate pathway (Fig. 4B). The disturbance of bioenergetics via mitochondrial dysfunction is a well-documented cause of CMT2^62–64^. Furthermore, mutations in the gene encoding pyruvate dehydrogenase kinase isoenzyme 3 (PDK3) cause CMT, a protein which links the glycolytic cascade to the Krebs cycle^65^. Based on our results, the involvement of glycolytic metabolism may be underappreciated in CMT2 pathophysiology. We speculate that glucose metabolism could contribute to CMT2 through reduced vesicular transport. Vesicular glycolysis is necessary and sufficient for the energy requirements of fast axonal transport (FAT)^66^. FAT depends on ATP generated by glycolysis, not by the mitochondria, and is reduced when glycolysis is perturbed^66^. FAT alteration is linked to multiple neurodegenerative diseases, and FAT restoration is likely to contribute to neuronal protection^67^.

The HSP-dominant modules were enriched for immune-related pathways: Epstein-Barr virus (EBV) infection, Herpes simplex (HSV) infection, and Antigen processing and presentation. EBV and HSV are members of the *Herpesviridae* family and have large, complex KEGG pathways which involve multiple sub-pathways including, for example, antigen processing and presentation. For a more detailed understanding of which EBV and HSV processes were most involved, we investigated the GO BP terms of the genes involved in the EBV and HSV pathways. The EBV pathway genes were enriched for membrane organization, protein targeting, and protein insertion into the mitochondrial membrane during apoptosis induction. The HSV pathway genes were enriched for transcription from the RNA polymerase II promoter. These results implicate antiviral mechanisms within HSP pathogenesis. Growing evidence documents the important roles of the mitochondria in antiviral immunity, including participation in signaling cascades and inflammation activation^68,69^. Since mitochondrial dysfunction is known to cause HSP, mitochondrial involvement may link HSP to innate immune responses^70^. Interestingly, EBV and human herpesvirus 6 (HHV-6) have been associated with multiple sclerosis (MS)^71^. HSP and MS both cause axonal loss of the corticospinal tract, share clinical similarities, and have concurred within families^72–74^.

## Concluding Remarks

In this study, we extracted insights about the spectrum of inherited axonopathies through the application of network-based tools to publically available protein interaction data. Starting with lists of known CMT2 and HSP disease genes, we determined significantly connected disease proto-modules from the global human interactome. The CMT2 and HSP proto-modules were topologically overlapping, supporting the consideration of these diseases as a related spectrum. We show the inherited axonopathies have an unclear (neither overlapping nor separated) relationship with ALS. These relationships will be elucidated as the molecular etiology of motor neuron diseases is further understood. We found a significant overlap between HSP and inherited ataxias, which is consistent with the ataxia-spasticity disease spectrum, while CMT2 was significantly separated from the ataxias^44^. This demonstrates the utility of network-based approaches in understanding the relationships between spectrums of closely related diseases. Each proto-module was expanded to identify additional proteins likely involved in disease pathogenesis, thus increasing the power of pathway analysis and providing candidate disease genes. We show that the inherited axonopathies are enriched for interactions with ribosomal proteins, which were recently shown to be preferentially localized to the axonal compartment of motor neurons^39^. Pathway analysis revealed biological processes enriched for each axonopathy independently, such as glucose metabolism for CMT2 and innate immunity for HSP, as well as for general axonopathies, such as new signaling pathways. Additionally, the identified candidate disease genes may guide exploration into drug target identification^71,75,76^. Sixty of the identified DIAMOnD proteins are present on the updated list of druggable genes: 49 DIAMOnDs may be targeted by a small molecule, 18 DIAMOnDs may be targeted by a biotherapeutic (monoclonal antibody/enzyme or other protein), and 1 DIAMOnD is involved in absorption, distribution, metabolism, and excretion of a compound^76^. Follow-up functional studies will be required to evaluate the potential involvement of DIAMOnD proteins in disease and their therapeutic potential. Overall, our study provides useful insights into inherited axonopathies relationships and disease mechanisms, and demonstrates the opportunities available through network-based approaches.

## Supplementary information

Supplemental Figures

Supplemental Table 1

Supplemental Table 2

Supplemental Table 3

## Data Availability

The datasets generated during and/or analysed during the current study are available from the corresponding author on reasonable request.
