# Supplementary material for: A network biology approach to unraveling inherited axonopathies
Source: Sci Rep. 2019 Feb 8;9:1692. doi: 10.1038/s41598-018-37119-z (PMC6368620; doi:10.1038/s41598-018-37119-z)
Supplement: Supplementary file 1 — Supplemental Figures [file 41598_2018_37119_MOESM1_ESM.pdf]

A network biology approach to unraveling inherited axonopathies

Dana M. Bis-Brewer[1], Matt C. Danzi[1], Stefan Wuchty[2], *Stephan Züchner[1]

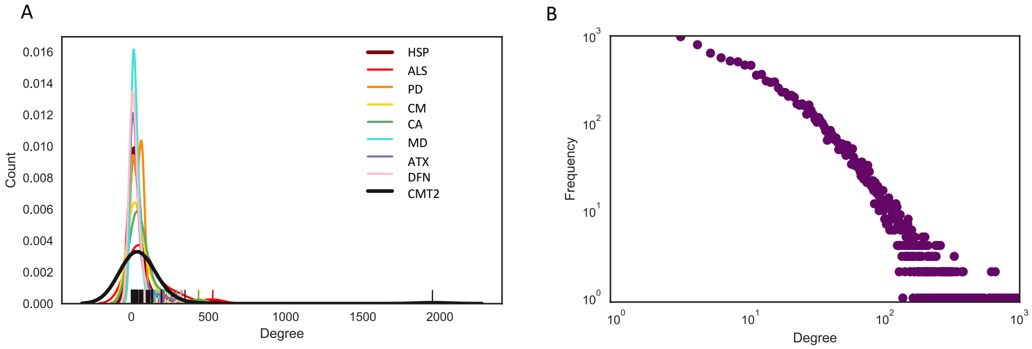

Supplemental Figure 1. Degree distributions of nodes from filtered HIPPIE PPI network. A) Degree distribution of disease genes interrogated for network localization (HSP: hereditary spastic paraplegia, ALS: amyotrophic lateral sclerosis, PD: Parkinson's disease, MD: muscular dystrophy, ATX: hereditary ataxia, CMT2: axonal Charcot-Marie-Tooth). B) Global degrees of nodes within the filtered network demonstrating a power law distribution expected from a scale-free network.

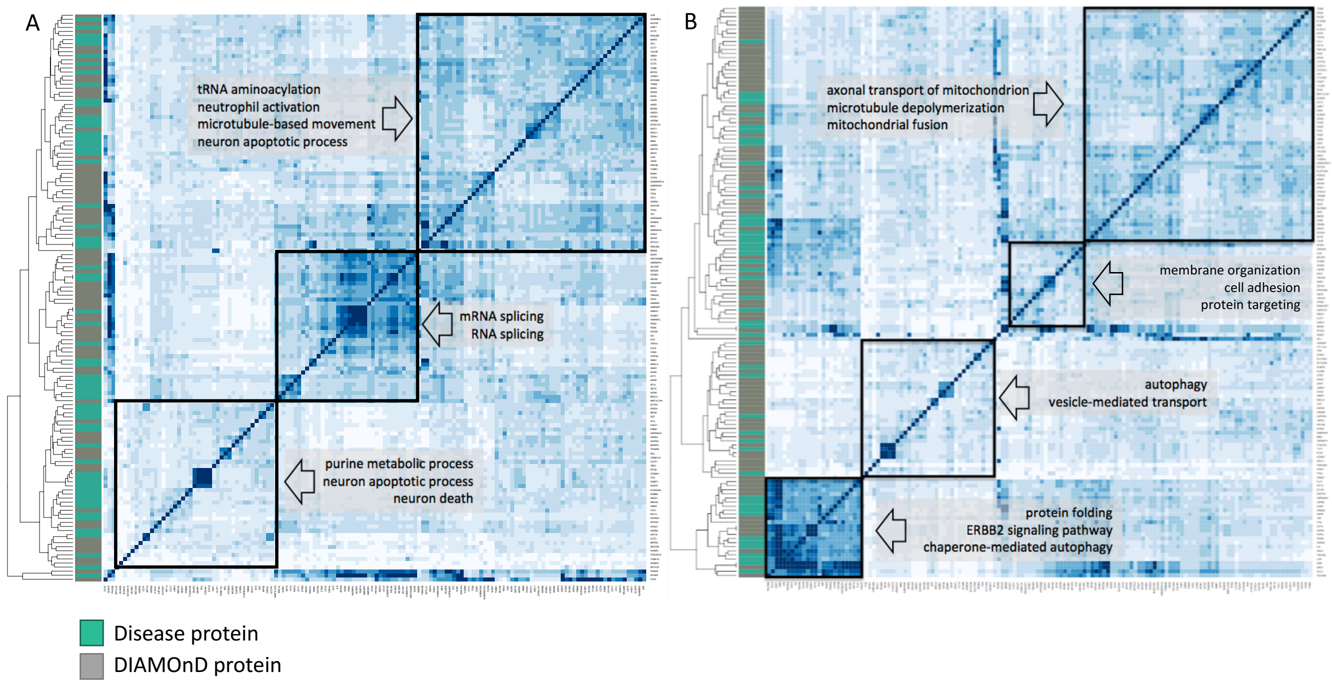

Supplemental Figure 2. Biological validation of inherited axonopathy DIAMOnDs. (A: CMT2, B: HSP). To limit the incorporation of false positives into each inherited axonopathy module, the DIAMOnD proteins were evaluated for biological evidence by comparing Gene Ontology Biological Process (GO BP) terms of the DIAMOnD proteins to the GO BP terms of the known disease proteins. Heatmaps (A & B) display the Wang semantic similarity of Gene Ontology Biological Process terms between disease gene (teal bars) and DIAMOnD gene (gray bars). To understand the underlying biological function of each identified cluster, we performed GO BP Over Representation Analysis (ORA) of the proteins within each cluster (annotated on each heatmap). Taken together, these results indicate that expanding the proto-modules leads to the identification of proteins that are functionally similar to disease proteins and are involved in biological processes that are relevant to the disease.

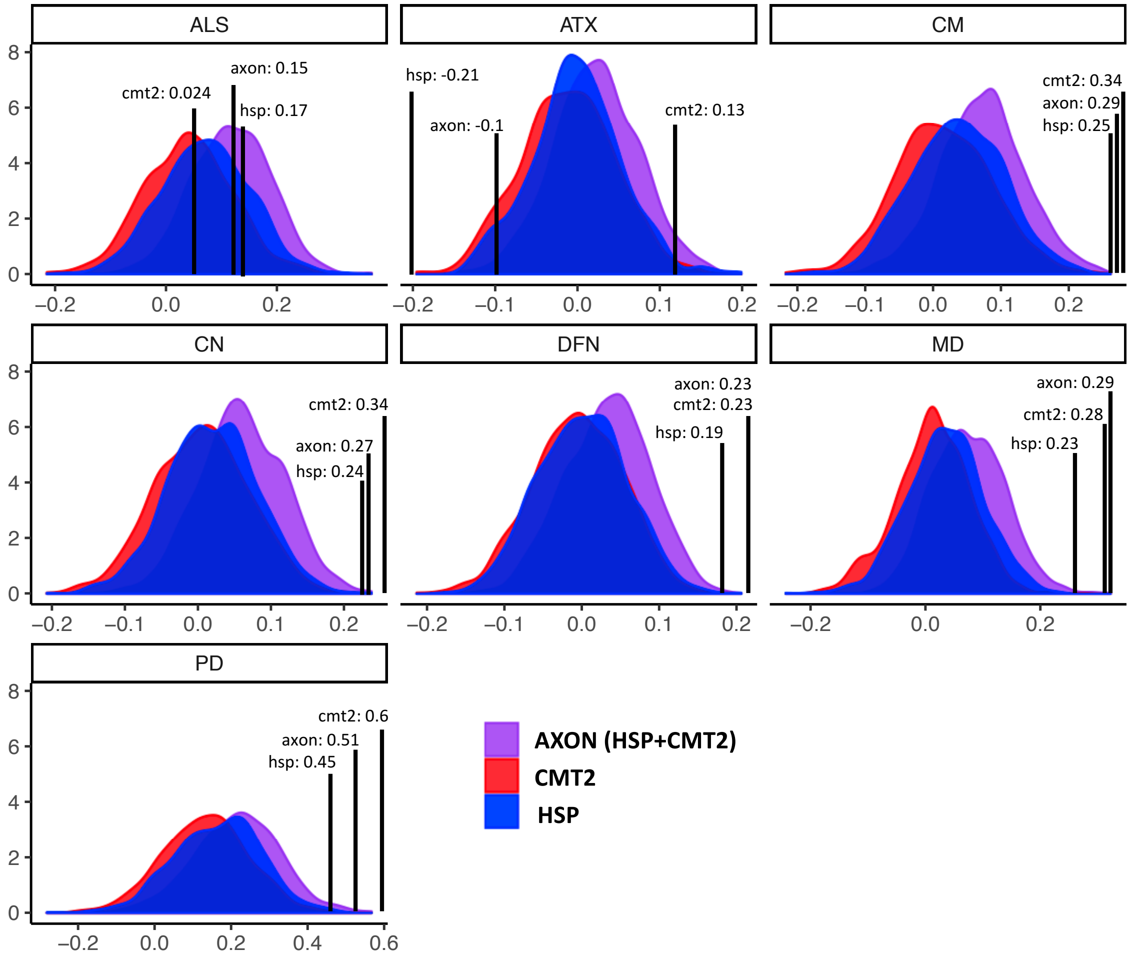

Supplemental Figure 3. Network distances between disease proto-modules. Disease modules that are located topologically adjacent to each other within the global network will likely share proteins, interactions, and pathways involved in disease pathogenesis. We explored the topological relationships between diseases by comparing the observed shortest distance between disease sets (annotated in black) to the expected network distances (density plot) for combined Inherited Axonopathies (HSP&CMT2: purple), Charcot-Marie-Tooth type 2 only (CMT2: red), and Hereditary Spastic Paraplegias only (HSP: blue) based on random gene sets.

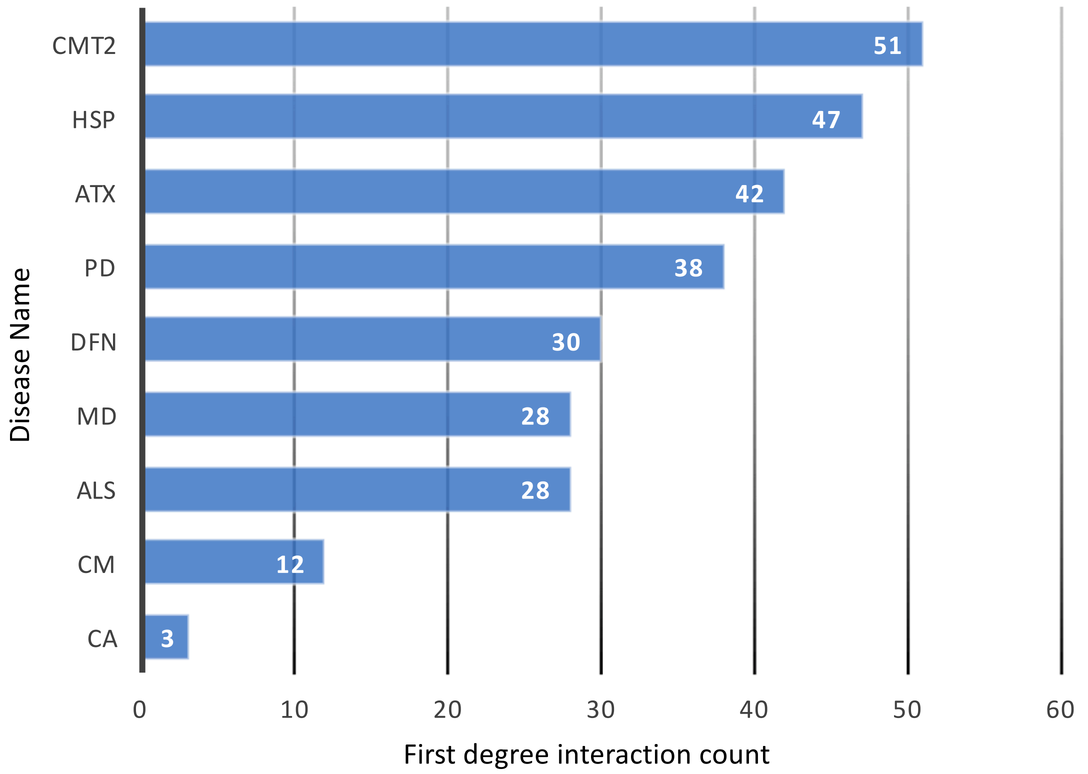

Supplemental Figure 4. DIAMOnD ribosomal protein interactions with disease seed proteins. To verify that ribosomal protein interactions are not an artifact of the HIPPIE network, we expanded each comparison disease proto-module and quantified the first degree interactions between disease seed proteins and ribosomal DIAMOnD proteins (Charcot-Marie-Tooth type 2: CMT2; Hereditary Spastic Paraplegia: HSP; Cancer: CA, Deafness: DFN, Cardiomyopathy: CM, Muscular Dystrophy: MD, Parkinson's Disease: PD, Amyotrophic Lateral Sclerosis: ALS, and Hereditary Ataxia: ATX).
